# Collaborative Drug Therapy Management: Case Studies of Three Community-Based Models of Care

**DOI:** 10.5888/pcd12.140504

**Published:** 2015-03-26

**Authors:** Margie E. Snyder, Tara R. Earl, Siobhan Gilchrist, Michael Greenberg, Holly Heisler, Michelle Revels, Dyann Matson-Koffman

**Affiliations:** Author Affiliations: Margie E. Snyder, Purdue University, Indianapolis, Indiana; Tara R. Earl, Michael Greenberg, Michelle Revels, ICF International, Inc, Atlanta, Georgia; Holly Heisler, ICF International, Inc, Cambridge, Massachusetts; Dyann Matson-Koffman, Office of the Associate Director for Science, Centers for Disease Control and Prevention, Atlanta, Georgia. Siobhan Gilchrist is also affiliated with IHRC, Inc, Atlanta, Georgia. At the time the study was conducted, Ms Gilchrist was affiliated with Columbus Technologies and Services, Inc, Atlanta, Georgia; Ms Heisler was affiliated with ICF International, Inc, Atlanta, Georgia; and Dr Matson-Koffman was affiliated with the Division for Heart Disease and Stroke Prevention, Centers for Disease Control and Prevention, Atlanta, Georgia.

## Abstract

Collaborative drug therapy management agreements are a strategy for expanding the role of pharmacists in team-based care with other providers. However, these agreements have not been widely implemented. This study describes the features of existing provider–pharmacist collaborative drug therapy management practices and identifies the facilitators and barriers to implementing such services in community settings. We conducted in-depth, qualitative interviews in 2012 in a federally qualified health center, an independent pharmacy, and a retail pharmacy chain. Facilitators included 1) ensuring pharmacists were adequately trained; 2) obtaining stakeholder (eg, physician) buy-in; and 3) leveraging academic partners. Barriers included 1) lack of pharmacist compensation; 2) hesitation among providers to trust pharmacists; 3) lack of time and resources; and 4) existing informal collaborations that resulted in reduced interest in formal agreements. The models described in this study could be used to strengthen clinical–community linkages through team-based care, particularly for chronic disease prevention and management.

## Introduction

In collaborative drug therapy management (CDTM), qualified pharmacists working in the context of a defined protocol are permitted to assume professional responsibility for performing a full scope of services: assessing patients; ordering drug therapy–related laboratory tests; administering drugs; and selecting, initiating, monitoring, continuing, and adjusting drug regimens (1). Authority for CDTM is generally incorporated into a state’s pharmacy practice act in the sections describing pharmacists’ scope of practice.

Pharmacist–provider collaborative practice agreements (CPAs), such as CDTM, are a strategy for expanding the pharmacist’s role in team-based care with other providers and improving health outcomes. CPAs can link patient care provided in traditional clinical settings with pharmacist care in community-based settings. CPAs emerged in the 1960s (2) and are now legally enabled in most states; however, the range of services authorized under each state’s practice act varies (3).

Pharmacist patient care services provided through CPAs have been shown to improve patient outcomes for diabetes, hypertension, anticoagulation, and other chronic diseases (4–6). The 2014 Community Preventive Services Task Force (Task Force) recently issued recommendations showing strong evidence for team-based care involving pharmacists and nurses to improve hypertension control and other chronic disease risk factors (7). Despite the noted benefits, pharmacists (particularly in community settings) are not routinely providing CDTM (8), although they may be collaborating informally with physicians to make drug therapy recommendations. One increasingly common opportunity for this informal collaboration is the use of medication therapy management (MTM), a required benefit for select Medicare Part D beneficiaries (9–11). In MTM as most commonly defined, a pharmacist reviews a patient’s medication regimen and must suggest changes to the prescribing physician for approval, rather than make any changes independently. This activity is permitted in any pharmacist’s scope of practice. CDTM takes this relationship a step further by enabling the pharmacist to make independent drug therapy changes under a protocol that may enhance the efficiency of the pharmacist and health care delivery.

There are more than 60,000 community-based pharmacies in retail settings (supermarkets, chain drug stores, and independent pharmacies), and approximately 39% of federally qualified health centers (FQHCs) have onsite pharmacies across the United States (12,13). The Centers for Disease Control and Prevention (CDC) funded this study to understand how CPAs, such as CDTM for hypertension management, are implemented in community pharmacies and to explore ways for more pharmacists to provide CDTM. The objectives of this study were to understand how CDTM practices were implemented in 3 community settings and to identify common and unique facilitators and barriers to implementing CDTM.

## Methods

### Case study site selection and inclusion criteria

We selected sites from states with scope of practice laws authorizing pharmacists to perform CDTM in any practice setting for a broad array of health conditions. We consulted with experts in MTM, CDTM, and collaborative models in pharmacy settings and reviewed the literature (14–17) to understand how CPAs might be implemented in different practice settings and to identify potential study sites. Three criteria emerged as the primary considerations for final site selection: duration, scope, and reach. Therefore, we sought a variety of sites, including some that were newly implemented (vs experienced), some that offered limited services (vs the full scope of CDTM services authorized by law), and some that reached a broad and diverse population (vs a more limited homogenous population).

We identified community pharmacies on the basis of expert recommendations and our literature review and contacted 10 sites. Each site received an email explaining the study and an invitation to participate. We excluded 5 sites for these reasons: CDTM services were not implemented because of the time and resources required, the contractual language of CPAs was considered prohibitive, or CDTM reimbursement mechanisms were lacking; 1 site declined; and another site did not respond. Ultimately, we used a combination of the criteria to select 3 sites that had CPAs in place: El Rio Community Health Center, Osterhaus Pharmacy, and Kerr Drug.

The research protocol was approved by the ICF International (ICF) institutional review board. CDC and Purdue University deferred to ICF.

### Recruitment

We worked with each site’s point of contact to identify potential key informants. Participants had to provide signed informed consent, be aged 18 years or older, and be comfortable speaking in English.

A purposive sample of potential informants at each site was recruited by email. To meet the study objectives, 9 key informants were recruited to share experiences on CDTM implementation. Three informants were recruited from each site: a pharmacist, a physician, and 1 other (eg, a pharmacy resident or administrator). Participants were not remunerated.

### Data collection

Case studies included key informant interviews and onsite observations. Before the site visits, interviewers completed a half-day training. Site visits took place during May through July 2012. Two study team members traveled to each site to conduct the interviews during a day-and-a-half visit. Because of the inability to schedule an in-person visit, we conducted telephone interviews for the Kerr Drug site. Interviews lasted about an hour and were audio recorded with consent.

A semistructured interview guide (available on request from the authors) was developed to focus on 6 topics: 1) CPA/CDTM policy implementation (eg, describe your CDTM policy and related guidelines for compliance with state law), 2) stakeholders, 3) effects of CPA/CDTMs on practice, 4) evaluation, 5) reimbursement, and 6) lessons learned and recommendations for implementing CPA/CDTM.

### Description of case study sites

#### El Rio Community Health Center (El Rio)

El Rio, an FQHC, is the largest provider of medical and dental services to uninsured and Medicaid-covered populations in Pima County, Arizona. Of 76,190 patients seen in 2011, 76% had an income at or below the federal poverty line, 48% received Medicaid, 28% were uninsured, 13% had private insurance, and 8% were Medicare recipients. El Rio serves a large Hispanic and Native American patient population, many with diabetes. El Rio has extensive experience implementing CDTM; its on-site pharmacists began entering into CPAs with El Rio providers in 2000. In 2012, approximately 800 patients received CDTM services, mostly for diabetes. The CDTM protocols also cover hypertension, hyperlipidemia, asthma, and other conditions. CPAs authorize pharmacists to assess patients, review medication regimens, adjust medications in approved drug classes, and perform specified examinations (eg, foot examinations) as well as patient drug reviews for medications that require monitoring, such as anticoagulation therapy. El Rio bills for CDTM services through Medicare Part D and for diabetes CDTM services as an accredited site for diabetes self-management training.

#### Osterhaus Pharmacy

Osterhaus Pharmacy is an independent, community pharmacy in the largely white (95%) rural community of Maquoketa, Iowa. Osterhaus Pharmacy serves approximately 6,500 patients annually, offering various patient care services. Approximately 60% of patients have diabetes, hypertension, and/or hyperlipidemia. In 2012, about 13% of patients received Medicaid and 42% were Medicare beneficiaries. Since 2000, the pharmacy has implemented limited elements of CDTM with a family medicine and emergency medicine group practice: a medication substitution and an immunization protocol. Although Osterhaus Pharmacy has extensive CDTM experience, this model is less comprehensive than authorized by Iowa law. For example, the CPAs operate under limited conditions, such as medication substitution based on patient insurance or influenza vaccination criteria, or the CPAs limit the scope of their services (ie, preclude modification of drug dosages based on laboratory or physical findings). In addition, the pharmacy and medical practice collaborate informally by providing MTM and other services.

#### Kerr Drug

Kerr Drug, a regional pharmacy chain, operates several retail stores in North Carolina that serve a varied patient population. Since 2007, Kerr Drug has provided MTM services to Medicare Part D beneficiaries. Although state law allows clinical pharmacist practitioners (CPP) (a distinct pharmacist credential that designates pharmacists practicing under a CPA) to perform a broad array of CDTM services, Kerr Drug recently implemented limited elements of CDTM. The Chapel Hill location completed a pharmacogenetic feasibility study involving a CPP arrangement with the primary investigator for the pharmacist to order genetic tests from a laboratory. To perform any other CDTM services, the CPP would need to collaborate with each study participant’s primary care provider. Kerr Drug’s feasibility study was funded by the University of North Carolina and grants.

### Analysis approach

Data analysis involved audio recorded debriefs by interviewers within a week of each site visit and a review of the recordings by 2 site visitors. Excel software (Microsoft Corp) was used to select salient quotations. Thematic categories guided by the case study questions (ie, key features, barriers, facilitators, and lessons learned) were selected and discussed for and across each site. Results were synthesized and sent to at least 1 key informant from each site for feedback.

## Results

### Key features of CDTM policy implementation

The elements of CDTM used at each site varied (Table 1).

**Table 1:** Elements of Collaborative Drug Therapy Management (CDTM) Offered at 3 US Sites, May–July 2012

CDTM Element	Case Study Site		
	El Rio Community Health Center^a^	Kerr Drug^b^	Osterhaus Pharmacy^c^
Perform patient assessments	X	X	X
Order drug therapy–related laboratory tests	X	X	
Administer drugs	X		X
Monitor and continue drug regimens^d^	X	X	X
Select, initiate, and adjust drug regimens^d^	X		X

a All elements authorized pursuant to Arizona Revised Statutes §§32–1901, 32–1970 and 32–1974 (AZ Admin Code R4–23-421 to R4–23-429 - Sections R4–23-421 - §R4–23-429 repealed by final rulemaking at 17 A.A.R. 2600, effective February 4, 2012 [Supp. 11–4]).

b All elements authorized pursuant to North Carolina General Statutes §§ 90–18(c)(3a) and 90–18.4 and North Carolina General Statutes §§90 85.1 et seq, and NC Admin Code tit 21 §46 .3101.

c All elements authorized pursuant to Iowa Code §155A.3 and 155A.44 and IA Admin Code tit 657 §8.2 & §8.34 and IA Admin Code tit 653 §13.4.

d Pursuant to specific boundaries of the CDTM agreement.

### Key barriers to CDTM policy implementation

Key barriers to CDTM policy implementation raised by pharmacists and physicians across the 3 sites included a lack of reimbursement mechanisms for CDTM services, difficulty establishing trusting relationships with providers, and the time and resources needed to perform CDTM patient care services (Table 2). Respondents reported that a key reason for not entering into CPAs was that pharmacists were not recognized as providers under federal law and, therefore, unable to bill for services. Physicians reported that many of their physician colleagues were initially hesitant to relinquish control of their patients’ drug therapy to pharmacists, particularly if they do not practice at the same location. However, it was reported that the distrust wanes over time. In addition, each state has different requirements, such as residency training or continuing education, for pharmacists to be eligible to engage in CDTM (1,3). The pharmacists reported that the application costs for various certifications and the time needed to devote to continuing education requirements can be burdensome. Finally, Kerr Drug and Osterhaus Pharmacy pharmacists achieved a great deal of collaborative patient care by making therapeutic recommendations (ie, MTM) without entering into CPAs. Their reasons for preferring informal collaborations to CPAs included the time and logistics required to create CPAs and the limited scope of diseases or medications that may be included in a CDTM protocol. In summary, compensation for these services was identified as a barrier, so in some cases, the disadvantages of the required time and logistics for CPAs outweighed the perceived benefits.

**Table 2:** Key Barriers to Collaborative Drug Therapy Management (CDTM) Policy Implementation at 3 US Sites, May–July 2012

Barrier	Case Study Site		
	El Rio Community Health Center	Kerr Drug	Osterhaus Pharmacy
Lack of reimbursement for CDTM services	X	X	X
Provider hesitation to trust pharmacists to deliver expanded services through CDTM	X	X	X
Lack of administrative resources and time	X	X	X
Proliferation of informal collaboration		X	X
Limited physical space to provide care	X		
Changing laboratory infrastructure		X	
Difficulty in building relationships with providers		X	
Limited access to patients’ clinical information			X
Patient challenges (eg, walk-in appointments, scheduling additional care)			X

### Key facilitators to CDTM policy implementation

The pharmacists and physicians interviewed at all 3 sites reported that physician buy-in, affiliation with an academic partner (eg, college of pharmacy), and having well-trained pharmacists on staff facilitated their ability to implement CDTM (Table 3). Pharmacist and provider collaboration — even on an informal or limited basis — helped solidify working relationships and increased provider buy-in over the long-term. Informants found that after providers began to experience the benefits of CDTM and other avenues of collaboration, they were more apt to collaborate.

**Table 3:** Key Facilitators to Collaborative Drug Therapy Management (CDTM) Policy Implementation at 3 US Sites, May–July 2012

Facilitator	Case Study Site		
	El Rio Community Health Center	Kerr Drug	Osterhaus Pharmacy
Increased stakeholder buy-in	X	X	X
Use of academic partnerships or pharmacy students or residents	X	X	X
Adequately trained pharmacists	X	X	X
Organizational culture of collaboration	X		X
Fewer regulatory or legal restrictions for engaging in CDTM agreements	X		
Broadly written CDTM protocols for providing patient care	X		
Patient satisfaction with receiving care from a pharmacist	X		
Sufficient space for patient counseling			X

Each site reported facilitators unique to their setting. At El Rio, the pharmacists widely disseminated reports describing positive patient outcomes, which helped to increase support for the collaborative care model. In addition, El Rio’s chief clinical pharmacist worked methodically to build relationships with newly employed officers, administrators, and providers. These relationships increased the number of patients referred to El Rio’s pharmacists and strengthened support among all stakeholders (eg, physicians). El Rio informants reported that a recent amendment to the state pharmacy act made engaging in CDTM less burdensome than in previous years. Finally, El Rio’s CDTM protocols are written broadly to give pharmacists substantial freedom in choosing how they care for patients.

Kerr Drug informants reported that the introduction of entry-level doctors of pharmacy (PharmDs) into the pharmacy profession contributed to the willingness of physicians to work collaboratively with pharmacists because of the rigorous scientific and clinical training involved in attaining the PharmD degree and because graduating physicians gain exposure to PharmD students during medical training. Furthermore, Kerr Drug has offered a pharmacy residency program for more than 12 years, and it serves as a training site for students. These factors increased interactions among Kerr Drug pharmacists and providers and raised providers’ support for and trust of the pharmacy profession. Similarly, the Osterhaus residency program was a critical part of collaborative relationships with providers. Osterhaus Pharmacy informants also mentioned having enough physical space to provide privacy for pharmacist–patient consultations. Finally, greater use of MTM via Medicare Part D and North Carolina’s CheckMeds program, which provides free pharmacist MTM services to beneficiaries enrolled in Medicare prescription drug plans, made it easier for pharmacists to enter into CPAs because providers realized the advantages of working closely with pharmacists.

## Discussion

To our knowledge, this is the first study of its kind to examine real-life examples of CDTM implementation in various community settings. Our results demonstrate that models for CDTM can be tailored to the needs of the pharmacists, the practitioners, and the patients they serve on the basis of the level of trust, training, and familiarity among practitioners. Even when state law allows practitioners and pharmacists to determine the scope of CDTM services (1,3), compensation mechanisms and legal requirements for certification and training and existing informal collaborative relationships limited pharmacists’ options or interest in expanding the array of CDTM elements offered.

A consortium, which was convened by the American Pharmacists Association Foundation (APhAF) and charged with developing strategies to advance pharmacist patient care services, identified 7 principles for optimizing the role of pharmacists in patient care (18). These principles resonate with the emergent themes from these case studies. The consortium reported that successful collaborations are established mutually by the collaborating health professionals on the basis of trust and demonstrated competence in a regulatory context that allows practitioners to establish the scope of the agreement (18). A 2011 survey of pharmacists also found that trustworthiness and professional interaction are predictive of established collaborative care relationships with physicians, whereas trustworthiness and role specificity are predictive of newly established collaborations (19). Each site reflects different levels of maturity in CDTM, and establishing trust over time through repeated professional interactions and the demonstrated value of pharmacist patient care services was a critical factor.

Informal collaboration between pharmacists and providers established trust and added value to patient care services, but it also resulted in some pharmacists reporting little need to enter into CPAs to perform more advanced patient care services, particularly given the logistics of these agreements and the limited compensation for CDTM services. Even though the case studies were conducted in states with permissive scope of practice laws that allow the practitioners to set the terms of the CPAs, some administrative and procedural legal requirements affected the study participants’ capacity to engage in CDTM, primarily because the costs and time commitment were considered burdensome.

One of the most common challenges for pharmacists and pharmacies reported by the sites and supported in the literature is the lack of sustainable compensation mechanisms (8,18,20). The APhAF consortium reported this challenge and stated that a scalable, sustainable, and financially viable business model is necessary for the successful implementation of pharmacist patient care services (18). Giberson et al describe several federal and state CDTM models that are successful because pharmacists are compensated for the patient care services they provide, but they explain that the private sector has yet to incorporate these models, in part because pharmacists lack recognition as providers under federal law (8). Furthermore, a survey of pharmacist clinicians practicing CDTM in 2 states suggested that CDTM is a business loss: respondents billed on average $6,500 per month for their services, far less than the average cost of hiring a pharmacist clinician (20). This highlights potential compensation challenges, even when states have tried to reduce financial barriers to expanding the provision of pharmacist services. These case study sites are funded by grants and private and public payer reimbursement for some services, including Medicare Part D and immunization fees, but not all services. A lack of sustainable compensation for pharmacist patient care services and the need for recognition of pharmacists as providers were reported across the sites. Therefore, although evidence indicates that expansion of pharmacists’ roles through CDTM could greatly benefit public health (4–6,8), new compensation models are needed for individual practices to implement CDTM.

The Task Force recommendations for team-based care involving pharmacists and nurses to improve cardiovascular disease risk factors underscore the results of this study. The Task Force recommendations are based on recent literature summarizing examples of collaborative models of hypertension management, including models that involved pharmacist interventions. Notably, the Task Force found larger improvements in hypertension control when pharmacists were team members, and medication adherence was greater when team members could change antihypertensive medications independent of or with approval of the primary care provider. However, the Task Force noted the need for appropriate reimbursement mechanisms for team members that may improve the perceived “benefit to barrier” ratio reported here and encourage more pharmacists to participate in hypertension control CPAs as an alternative to informal collaborations with providers (7).

This study has several limitations. The sites selected might not be representative of all community-based CDTM practices. For example, Kerr Drug pharmacists served patients participating in a research study. El Rio is an FQHC where pharmacists, physicians, and other providers practice in a relatively closed setting. Furthermore, time and resource constraints prevented full transcription of audio recordings and analysis via formal data-coding procedures. Finally, the number of sites was small. It is not known whether visiting more sites would have identified additional themes.

As health care delivery systems increasingly adopt models of team-based care, such as CDTM, business and practice models and policies need to adapt accordingly. Although pharmacist interventions positively affect hypertension and other chronic diseases, these case studies highlight challenges and varying approaches to implementing CDTM. Pharmacists, other providers, and decision makers can use these findings when considering collaborative practice models to expand the pharmacist’s role in team-based care, link patient care in clinical settings with community-based services, and improve health outcomes. Results of this study will be available as guidance documents on CDC’s website.
