# Erratum, Vol. 12, March 26 Release

**DOI:** 10.5888/pcd12.140504e

**Published:** 2015-07-23

Two funding sources were inadvertently omitted from the Acknowledgments section of the article “Collaborative Drug Therapy Management: Case Studies of Three Community-Based Models of Care,” which was released on March 26, 2015. The following information was added to the article on July 7, 2015, and appears online at www.cdc.gov/pcd/issues/2015/14_0504.htm: “Dr Snyder’s effort was partially supported by grant no. KL2RR025760 from the National Institutes of Health (Anantha Shekhar, principal investigator) and by grant no. K08HS022119 from the Agency for Healthcare Research and Quality (M.E.S., principal investigator).”

